# Coronavirus Disease 2019-Associated Mucormycosis Diagnosed by Fine-Needle Aspiration Cytology

**DOI:** 10.7759/cureus.21990

**Published:** 2022-02-07

**Authors:** Radhika Narayan, Minakshi Mishra, Minakshi Gupta, Sangita Kamath, Riti Chitrotpala

**Affiliations:** 1 Pathology, Tata Main Hospital, Jamshedpur, IND; 2 Microbiology, Tata Main Hospital, Jamshedpur, IND; 3 General Medicine, Tata Main Hospital, Jamshedpur, IND; 4 Radiology, Tata Main Hospital, Jamshedpur, IND

**Keywords:** covid-19-associated mucormycosis, fine-needle aspiration cytology, aseptate hyphae, fungal culture, fungal hyphae, pulmonary mucormycosis

## Abstract

The temporal association of mucormycosis with coronavirus disease 2019 (COVID-19) has been termed COVID-19-associated mucormycosis (CAM). Because of its poor prognosis, early diagnosis and treatment are crucial. Although tissue samples for culture and histological evaluation are the cornerstones of diagnosis, the role of fine-needle aspiration cytology (FNAC) and cytologic findings are also significant. Here, we report a case of mucormycosis in a COVID-19-positive 34-year-old male diagnosed by FNAC and confirmed by fungal culture. To our knowledge, this is possibly the first and only case report of CAM diagnosed by FNAC.

## Introduction

Coronavirus disease 2019 (COVID-19)-associated mucormycosis (CAM) is the term used to describe the association of mucormycosis in COVID-19 patients that was rampant during the second wave of the pandemic. Indiscriminate use of corticosteroids, uncontrolled blood sugar levels, and viral lymphopenia are some key factors implicated in the development of mucormycosis in COVID-19 patients [1]. Although tissue biopsy and culture are essential to confirm the diagnosis of mucormycosis, the cytological findings obtained on fine-needle aspiration cytology (FNAC) are also important in early and reliable diagnosis, especially in a critical situation, as seen in our patient. This case report probably represents the first reported case of CAM diagnosed by FNAC and confirmed by fungal culture.

## Case presentation

**Figure 1:**
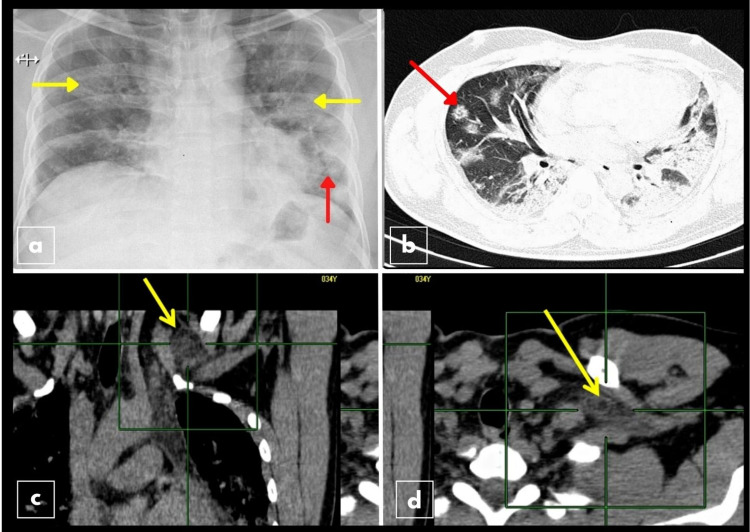
Radiological findings on chest X-ray and CT scan of the thorax. a: Chest X-ray, PA view: bilateral patchy ground-glass opacification in middle zones (yellow arrow) and left lower zone consolidation in the lung with air bronchogram (red arrow). b: Reverse halo sign (red arrow) on NCCT, characteristic of invasive mucormycosis. Coronal (c) and axial (d) NCCT image in soft tissue window showing fat stranding and hyperdensity in left subclavicular soft tissue (yellow arrows), suggesting the spread of inflammation from the lung. CT: computerized tomography; PA: posteroanterior; NCCT: non-contrast-enhanced computerized tomography

A 34-year-old male patient was admitted to our hospital with complaints of fever and difficulty in breathing for the past seven days. He was not a known diabetic or hypertensive. On presentation, he was conscious and afebrile, with SpO_2_ of 82% on room air. The reverse transcription-polymerase chain reaction test for severe acute respiratory syndrome coronavirus 2 was positive. Chest examination showed bilateral air entry. Additionally, cardiovascular system examination and abdominal examination were normal. His routine blood investigations showed hemoglobin of 15.3 g/dL, total leukocyte count of 8,300/mm^3^ with 81% neutrophils, and platelet count of 112,000/mm^3^. His C-reactive protein (CRP) level was 18.93 mg/dL, and his lactate dehydrogenase level was 725 U/L. His blood sugar levels were monitored in the ward and were not elevated. Serum ferritin level was 1,929 ng/mL. The radiological findings on chest X-ray and non-contrast-enhanced computerized tomography (CT) are shown in Figure 1.

**Figure 2:**
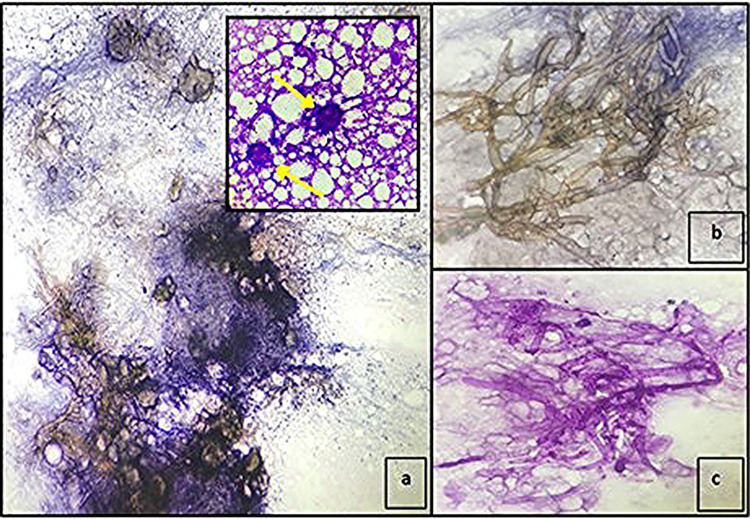
FNAC smears of left subclavicular swelling aspirate. a: Shows broad, right-angled branched aseptate hyphae of Mucor in a dirty necrotic background (PAP stain, 10×); inset: basophilic calcifications (yellow arrows) MGG stain, 40×. b: High-power view of Mucor hyphae (PAP stain, 40×). c: Hyphae of Mucor are PAS-positive (PAS stain, 40×). FNAC: fine-needle aspiration cytology; PAP: Papanicolaou stain; MGG: May-Grunwald-Giemsa; PAS: periodic acid-Schiff

The patient’s chest X-ray (Figure 1a) showed bilateral pneumonia and left lower zone opacities. The CT severity score on high-resolution CT was 20/25. Extensive bilateral ground-glass densities were noted involving approximately 75% of the lung parenchyma with areas of subpleural fibrosis and architectural distortion. He was shifted to the intensive care unit for non-invasive ventilation. On the eighth day of admission, he complained of swelling and pain in the neck with restricted movement. On examination, there was a diffuse, firm, non-tender swelling measuring 2 × 2 cm in the left supraclavicular area. FNAC of the supraclavicular swelling yielded a scanty, particulate, and mildly blood-mixed aspirate. Cytologic examination showed a dispersed population of neutrophils and dispersed aggregates of histiocytes in a necrotic background. The lymph node background was not seen. There were several branched aseptate fungal hyphae with some broad-based ends without spore formation (Figure 2).

**Figure 3:**
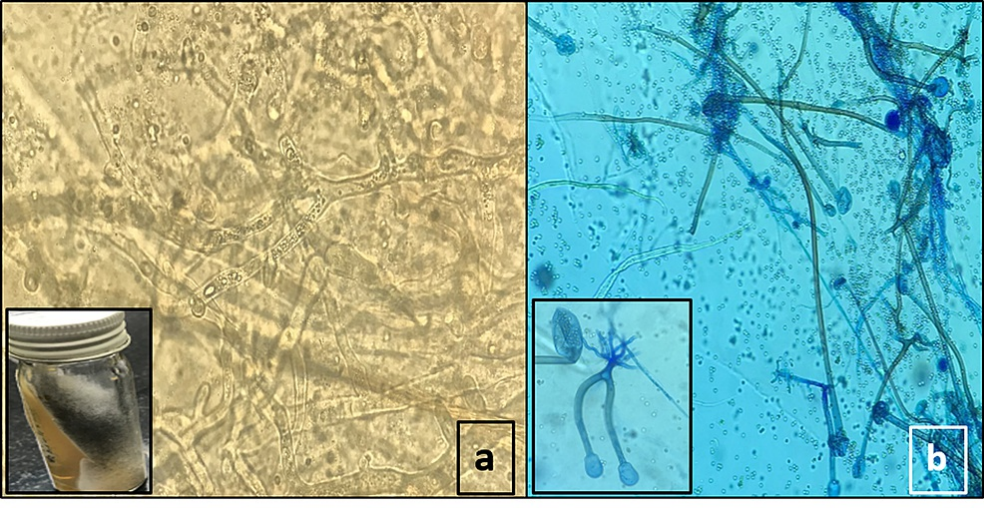
Findings of fungal culture and staining. a: 10% KOH mount preparation of aspirated material showing aseptate Mucor hyphae (40×); inset: blackish whitish colonies of Mucor in SDA medium. b: Hyphae and sporangiospores of Mucor in LCB-stained smears (10×); inset: magnified view of sporangiospores (40×). SDA: Saborarud dextrose agar; LCB: lactophenol cotton blue

May-Grunwald-Giemsa (MGG) stain showed some loose aggregates of basophilic amorphous material resembling calcification (Figure 2a, inset). Periodic acid-Schiff (PAS) stain highlighted the fungal hyphae. Overall, the FNAC findings suggested fungal inflammatory pathology consistent with CAM. There was no evidence of granulomatous inflammation or malignancy. The aspirate material was also examined under a 10% KOH mount and showed few aseptate broad-based fungal hyphae. The aspirate was also cultured in Sabouraud dextrose agar media and showed growth on the third day. Lactophenol cotton blue stain prepared from the growth showed several hyaline hyphae and sporangiospores (Figure 3).

In light of the above cytological and culture findings, the HRCT findings were reviewed. There were focal areas of the reverse halo sign in the right middle lobe region, which is specific for pulmonary mucormycosis (Figure 1b). There was also an extension into the anterior mediastinum along with focal areas of fat stranding in the left subclavicular region, suggestive of the spread of infection (Figure 1c). He was started on tablet posaconazole 300 mg twice a day on day one, followed by 300 mg once daily and injection dexamethasone 40 mg intravenously once daily. Liposomal amphotericin B (LAmB) could not be given as the patient could not afford treatment. Unfortunately, due to his economic constraints, the patient sought discharge against medical advice. On follow-up five months later, the patient reported that he had taken oral posaconazole 300 mg once daily for three months. At present, he is well and remains asymptomatic.

## Discussion

One of the most dreaded opportunistic infections associated with COVID-19 that has ravaged the world is mucormycosis which caused significant morbidity and mortality in India during the second wave of the pandemic. Mucormycosis is an angioinvasive fungal infection caused by a fungus of the order Mucorales. It is usually present in the environment and grows on wet surfaces and dead and decaying vegetable matter. Predisposing risk factors for mucormycosis include immunosuppression, chronic kidney disease, malignancy, neutropenia, increased serum iron, and, most importantly, diabetes mellitus with ketoacidosis [1].

Mucormycosis is classified into the following five forms depending on the site of infection: rhinocerebral, pulmonary, cutaneous, gastrointestinal, and disseminated type [2]. Pulmonary mucormycosis is the second most common form, accounting for 80% of the mortality due to delayed diagnosis and inadequate treatment. Pulmonary infections can spread to contiguous structures, such as the mediastinum, heart, chest wall, and pleura [3].

The first case of pulmonary CAM in a COVID-19 patient was reported by Pasero et al. [4] in December 2020, wherein the patient’s bronchial aspirate sample was used to grow and confirm the fungus. In a systemic review of the epidemiology and pathophysiology of CAM reported globally till June 21, 2021, Muthu et al. [5] reported 275 cases of CAM, of which 233 were from India and 42 from the rest of the world. Rhino-orbital mucormycosis and rhino-orbital-cerebral mucormycosis accounted for 89% of cases in India, while, globally, they accounted for 64% of cases. Pulmonary CAM was less frequent in India (7.3% vs. 21.4%). Similar findings were reported in two other systemic reviews of CAM by Singh et al. [6] and Pal et al. [7]. Although underlying diabetes was seen in 66.1% of CAM patients, 28.8% of patients presented with COVID-19 symptoms only [5]. Our patient had features of pulmonary CAM, with high-resolution CT showing extensive bilateral ground-glass densities, as well as focal areas of “reverse halo sign” noted in the right middle lobe region. A localized area of fat stranding in the left subclavicular region contiguous with fat stranding of the anterior mediastinum was noted, suggesting a possible pulmonary origin and spread to the anterior mediastinum. The “reverse halo sign” on the CT scan is specific to pulmonary mucormycosis and helps distinguish it from other fungal infections, especially aspergillosis (54% of pulmonary mucormycosis compared to 6% of those with invasive pulmonary aspergillosis) [8]. The CT findings of invasive pulmonary aspergillosis, a close mimic of pulmonary mucormycosis, include clusters of centrilobular nodules, consolidation, cavitary lesions, and pleural effusions. Treatment for mucormycosis includes LAmB administered intravenously at a dose of 5 mg/kg body weight infused over three hours in a 5% dextrose solution, followed by oral posaconazole 300 mg twice a day on day one, followed by 300 mg once daily for three to six months depending upon the response to therapy. Treatment of choice for invasive aspergillosis includes voriconazole and isavuconazole with LAmB as the first alternative, and posaconazole being used for salvage treatment.

The identification of fungal elements in histological sections and fresh clinical specimens, such as bronchoalveolar lavage and biopsy, confirm infection. Although site-specific biopsy and culture are important for the diagnosis of mucormycosis, guided FNAC also plays a crucial role in pulmonary CAM [9]. The utility of FNAC in the diagnosis of pulmonary lesions has been reported in several previous studies. In a study of pulmonary infections by image-guided FNAC in 42 immunocompromised patients, Sharma et al. found that the most common cytologic diagnosis was mucormycosis [10]. There are several case reports published in the medical literature of pulmonary mucormycosis diagnosed by FNAC [11-14]. The cytologic material in these cases was obtained by bronchial brush cytology or transthoracic needle aspiration and confirmed by fungal culture or biopsy with histopathology. To our knowledge, CAM diagnosed by FNAC has not been reported so far, and this case possibly represents the first case.

The morphological differences between Mucor and Aspergillus in the cytological and histopathological examination are important for accurate diagnosis and guiding the choice of treatment. On cytology, the hyphae of Mucor are quite variable in size, averaging 15-20 µm in diameter, are aseptate, and show right-angle branching. However, Aspergillus hyphae, measuring 3-6 µm, are septate and show regular, dichotomous, acute angle (45 degrees) branching. The tendency of the Mucor hyphae to fold and wrinkle gives them a ribbon-like appearance which is very helpful in identification. Another cytological finding seen in our patient was the presence of bluish calcific material on the MGG stain, possibly representing calcium oxalate crystals (Figure 2a; inset); these are known to be produced occasionally as a metabolic byproduct of Mucor infections [12]. On tissue biopsy, the Mucor hyphae are broad, ribbon-like, thin-walled, primarily aseptate, or pauci-septate hyphae that have irregular diameters, along with non-dichotomous, irregular branching, and accompanying tissue necrosis and fungal angioinvasion. In tissue sections, Aspergillus hyphae appear as septate filaments, 5-10 µm thick, branch at acute angles, and form fruiting bodies. Because it tends to invade blood vessels, areas of hemorrhage, infarction, and necrotizing inflammation are commonly seen [15].

We performed an extensive search of PubMed and Medline but did not come across any study reporting post-COVID-19 mucormycosis diagnosed on FNAC; hence, this case probably represents the first case of pulmonary CAM to be diagnosed by FNAC and confirmed by fungal culture. Pulmonary mucormycosis presenting with a neck mass is very uncommon, and the literature review revealed only one such uncommon case reported by Ukoha and Nguyen in 2021 [16]. The factors that helped our patient to recover from the deadly CAM were possibly the absence of any pre-existing comorbidities, such as diabetes and hypertension, combined with prompt FNAC-based diagnosis and management of mucormycosis.

## Conclusions

This case highlights the utility of FNAC in providing an early and accurate diagnosis of mucormycosis, especially in a critical situation like CAM, where the timely diagnosis is key to the successful management of a potentially serious and fatal condition. Although tissue biopsy confirmation is recommended, FNAC is very useful to obtain adequate diagnostic material, especially in pulmonary mucormycosis. The characteristic appearance of the fungal hyphae on aspirate smears and confirmation by fungal culture helps establish the diagnosis of mucormycosis in any suspected patient of CAM. The possibility that mucormycosis can also present as a soft tissue swelling, especially in COVID-19 patients, should be kept in mind and needs urgent attention.
